# RUNX2 facilitates aggressiveness and chemoresistance of triple negative breast cancer cells *via* activating MMP1

**DOI:** 10.3389/fonc.2022.996080

**Published:** 2022-11-18

**Authors:** Wentao Si, Xiaodan Xu, Lijuan Wan, Fengxu Lv, Wei Wei, Xiaojun Xu, Wei Li, Dabing Huang, Leisheng Zhang, Feifei Li

**Affiliations:** ^1^ Department of Pathophysiology, School of Basic Medical Sciences, Anhui Medical University, Hefei, China; ^2^ Department of Pathology, The First Affiliated Hospital of Anhui Medical University, Hefei, China; ^3^ Department of Oncology, The First Affiliated Hospital of Anhui Medical University, Hefei, China; ^4^ Department of Breast Surgery, The First Affiliated Hospital of Anhui Medical University, Hefei, China; ^5^ Department of Oncology, The Second Affiliated Hospital of Anhui Medical University, Hefei, China; ^6^ Department of Oncology, The First Affiliated Hospital of University of Science and Technology of China, Hefei, China; ^7^ Division of Life Sciences and Medicine, University of Science and Technology of China, Hefei, China; ^8^ Key Laboratory of Molecular Diagnostics and Precision Medicine for Surgical Oncology in Gansu Province & NHC Key Laboratory of Diagnosis and Therapy of Gastrointestinal Tumor, Gansu Provincial Hospital, Lanzhou, China; ^9^ Key Laboratory of Radiation Technology and Biophysics, Hefei Institute of Physical Science, Chinese Academy of Sciences, Hefei, China

**Keywords:** triple negative breast cancer, aggressiveness, chemoresistance, RUNX2, MMP1

## Abstract

Breast cancer remains the most common malignancy in women and constantly threatens the lives of patients worldwide. State-of-the-art renewal has indicated the involvement of RUNX-associated transcription factor 2 (RUNX2) in tumorigenesis and cancer progression, yet the detailed information during breast cancer is largely obscure. Herein, we took advantage of breast cancer cell lines and *in vivo* tumorigenicity test as well as multifaceted phenotypic analyses (e.g., RNA-sequencing, ChIP and qRT-PCR assay) to verify the pathogenic mechanism of RUNX2 in triple negative breast cancer aggressiveness and chemoresistance. Strikingly, the proliferation, migration, invasion and chemoresistance of resistant cell lines in triple negative breast cancer was effectively suppressed by RUNX2 silencing, and the *in vivo* tumorigenicity was significantly weakened as well. Furthermore, with the aid of transcriptomic and bioinformatic analyses, we found MMP1 was highly expressed in triple negative breast cancer (TNBC) and showed a strong correlation with the poor prognosis of the patients, which was consistent with the expression pattern of RUNX2. Finally, by conducting ChIP and qRT-PCR assessment, we verified that RUNX2 functioned *via* directly binding to the specific motifs in the promoter of MMP1 and thus activating the transcriptional process. Collectively, our data demonstrated the facilitating effect of RUNX2 during triple negative breast cancer progression by directly orchestrating the expression of MMP1, which supplied overwhelming new references for RUNX2-MMP1 axis serving as a novel candidate for breast cancer diagnosis and treatment.

## Introduction

Breast cancer is one of the most common malignancies and the leading cause of cancer related death in women, which also has drawn public-health concerns worldwide (1). Despite the dramatic progress in clinical practice, surgery and chemotherapy remain the major treatment options for breast cancer administration (2). As a particularly aggressive subtype, triple negative breast cancer (TNBC) has been regarded as the most challenging breast cancer without hormone receptors and human epidermal growth factor receptor 2 (HER2) gene amplification, which commonly results in greater metastatic potential, higher rates of relapse, together with shorter overall survival compared with other subtypes (3). Worse still, due to the high metastatic nature and tumor heterogeneity as well as drug resistance, breast cancer is still intractable for clinicians (4). Thus, there’s an urgent need to identify novel biomarkers and dissect the underlying molecular mechanisms of metastasis and drug resistance, which will provide novel therapeutic targets for drug development and the clinical treatment decisions of breast cancer patients.

RUNX2 is a transcription factor that has been reported essential for skeletal development, chondrocyte maturation and osteoblast differentiation (5–7). To date, literatures in the field have also suggested the involvement of RUNX2 in various malignant progression. For instance, He et al. and Li et al. recently indicated the aberrant phosphorylation and expression of RUNX2 in breast cancer invasion and gastric cancer metastasis, respectively (8, 9). Of note, Vishal and the colleagues further reviewed the potential dysfunction and regulation of RUNX2 in breast cancer mediated bone metastasis, yet the regulatory mechanism during the metastatic process and drug resistance in triple negative breast cancer was insufficiently disclosed (10).

For the purpose, we conducted multifaceted analyses both *in vitro* and *in vivo* to verify the dysfunction and the concomitant mechanism of RUNX2 in modulating proliferation, migration, invasion, and chemoresistance of breast cancer cells. Notably, with the aid of transcriptomic analysis and ChIP assay, we found that RUNX2 was adequate to regulating MMP1 expression by directly binding to the specific motifs in the promoter region. Compared to normal breast epithelium, triple negative breast cancer cells and tissues revealed higher level of MMP1 expression, which also showed perfect positive correlation with poor prognosis in breast cancer patients. Taken together, our findings highlighted the pathogenic mechanism of RUNX2-MMP1 axis in breast cancer, which further provided new insights into metastasis and chemoresistance of triple negative breast cancer.

## Materials and methods

### Cell lines

MDA-MB-231 and SUM-149 were purchased from the American Type Culture Collection (ATCC, USA), and MDA-MB-231 was cultured in high glucose DMEM medium (PM150210, Procell) supplemented with 10% fetal bovine serum (164210, Procell) and 1% penicillin and streptomycin (PB180120, Procell). SUM-149 was cultured in Ham’s F12 medium (L410KJ, BasalMedia) supplemented with 5% fetal bovine serum, 5µg/ml of insulin (UNITED LABORATORIES), and 1µg/ml of hydrocortisone (R011855, RHAWN). MDA-MB-231-Re epirubicin-resistant cell lines were established as previously described (11). All cells were cultured in 5% CO_2_, at 37°C.

### Lentiviral stable cell lines

MDA-MB-231-Re cells were seeded in six-well plates and incubated for 24 h. Lentivirus containing shRNA (shRUNX2, NC; MOI=10) was added when cell reached 20-35% confluency. Then, the supernatant was replaced with fresh medium after 10 h, followed by the observation of GFP fluorescence under a microscope at 72 h and stable line selection by puromycin addition. MDA-MB-231-Re cells with shRNA NC infection were used as a negative control.

Similarly, the RUNX2 over-expression (denoted as RUNX2) and the negative control (denoted as Vector) SUM-149 cell lines were generated by utilizing the aforementioned lentiviral strategy.

### SiRNA transfection

For RUNX2 interference with the specific siRNAs (siRUNX2#1, siRUNX2#2) (GenePharma), MDA-MB-231 cells were cultured in 60-mm dishes for 24 h and the medium was replaced with fresh medium containing 200 pmol siRNA and 10µl Lipo6000™ Transfection Reagent (C0526, Beyotime) for 5 h when cells reached 30-50% confluence. Then, the siRNA-Transfected MDA-MB-231 cells was cultured in fresh medium, and the efficiency of interference for RUNX2 (siRUNX2#1, siRUNX2#2) and negative mimic (NC) was verified 48 h later by utilizing the qRT-PCR assay.

### Cell counting kit-8 assay

To verify the cytotoxicity of the indicated anti-cancer drug at various concentrations, the cells were seeded in 96-well plates at a density of 5000 cells/well in 100 µl culture medium and incubated at 37°C, 5% CO_2_ for 24 h. Then, the cells were incubated with anti-cancer drug epirubicin (Pfizer) for 24 h, and 10 µl/well CCK-8 solution (GK10001, GlpBio) was added for another 2 h. Finally, the absorbance value was read at 450 nm by using a microplate reader (Thermo).

### RNA isolation and quantitative real-time PCR

Total RNAs were isolated by using TRIzol Reagent (CW0580S, CWBIO) according to the manufacturer’s instructions. Then, cDNAs were synthesized with the Reverse Transcriptase kit (E047-01B, Novoprotein) as we previously reported (12). After that, qRT-PCR analysis was conducted by using the SYBR Green Pro Taq HS premixed qPCR kit (AG11701-S, Accurate Biotechnology) and the ABI PRISM 7900 (Applied Biosystems). In details, an initial denaturation step of 95°C for 30s, followed by 40 cycles of 95°C for 5s and 60°C for 30s. The results were calculated by using 2^-ΔΔCt^ method. The primer sequences were shown in **Supplementary Table S1**. GAPDH was used as internal reference.

### Protein extraction and western blotting assay

Total protein was extracted with RIPA lysis buffer from the indicated cells, and the concentration was measured with the BCA Protein Assay Kit (P0012, Beyotime). The samples were subjected to 10% sodium dodecyl sulfate-polyacrylamide gel electrophoresis (SDS-PAGE) and proteins were transferred to a PVDF membrane (IPVH00010, Millipore). After that, the membranes were incubated with primary antibody overnight at 4°C and secondary antibody for 1 h at room temperature, respectively. Finally, the protein bands were developed by using an enhanced chemiluminescence detection reagent (D045, Bridgen) and imaged by using an Image Lab (BIO-RAD). Primary antibodies against RUNX2 (12556, CST, dilution 1:1000), MMP1 (DF6325, Affinity, dilution 1:1000), E-CADHERIN (20874-1-AP, Proteintech, dilution 1:5000), VIMENTIN (10366-1-AP, Proteintech, dilution 1:5000), and β-ACTIN (AF7018, Affinity, dilution 1:5000) were used. Three independent experiments were conducted.

### Transwell assay

Cells preconditioned in DMEM basal medium (without 10% FBS) for 12 h were seeded in the upper chamber of the 24-well plate transwell insert (353097, Corning) at a density of 10^5^ cells/well, and 600 µl of DMEM complete medium (with 10% FBS) were added to the lower chamber. Then, the cells were fixed with 10% formalin (YULU) for 20 min after incubation for 24- 48 h, following by staining with 0.1% crystal violet (C8470, Solarbio) for 20 min. After wiping the non-migratory cells in the inner side of the upper chamber, images of migrated cells were captured from three randomly selected fields under the microscope, and the number of cells in each field was calculated.

The invasion assay was similar to the migration assay, where the upper chamber was coated with matrix gel (356234, Corning).

### Plate cloning assay

Breast cancer cells were seeded in 6-well plates at 1000 cells/well, and cultured for 14 days at 37°C, 5% CO_2_. The cells were fixed with 10% formalin for 20 min, followed by staining with 0.1% crystal violet for 20 min. Typical colonies (foci > 100 µm) were counted. Three independent experiments were conducted.

### Soft agar colony formation assay

To evaluate the malignant proliferative capacity of the indicated breast cancer cells, we conducted the soft agar colony formation assay. Briefly, the bottom layer was composed of 1.2% low melting point agarose (BS144, Biosharp) for preventing cell adhesion, and the upper layer was made up of 0.7% low melting point agarose containing 2000 cells/well. After 14 days, the morphology and count of colonies were recorded under the microscope.

### Xenograft assay and histological analysis

5-week-old female BALB/c nude mice were applied for xenograft assay according to the Guide for the Care and Use of Laboratory Animals and were approved by the Experimental Animal Ethics Committee of Anhui Medical University (Approval No.: LLSC20210808). 5×10^6^ NC or shRUNX2-transduced MDA-MB-231-Re cells were subcutaneously injected into 5-week-old female BALB/c nude mice (N=4) in each group. Tumor long diameter (L) and short diameter (W) were measured every 3 days. The volume of the xenograft was calculated according to the formula: volume (mm^3^(=L×W^2^×0.5. Finally, the morphology and weight of the xenograft in nude mice in the indicated groups were recorded at day 33 of the xenograft assay. Besides, mice were sacrificed and tumors were resected for immunohistochemical staining.

### RNA-sequencing and bioinformatic analyses

For preparation of RNA-SEQ samples, total RNAs were isolated by using the TRIzol Reagent according to the manufacturer’s instructions. Then, the RNAs were sequenced by Personal Bio Company with the Illumina mRNA-Seq sample preparation kit and the Illumina Hiseq sequencing platform (Illumina, Inc.). The multifaceted bioinformatic analyses, including HeatMap, volcano plots, Gene Ontology (GO) and Kyoto Encyclopedia of Genes and Genomes (KEGG) were accomplished as we recently reported with several modifications (13).

### Chromatin immunoprecipitation and qRT-PCR assay

Cells were crosslinked with 37% formaldehyde and lysed in SDS buffer. Then, the DNA was fragmented by using sonication. For RUNX2 binding sites on MMP1 promoter, ChIP assay was performed as we previously reported (14).

The eluted DNA fragments were turned to qRT-PCR assay, and the values generated from immunoprecipitated samples were normalized to those of the corresponding input samples. Besides, qRT-PCR products were stored at -20°C in a refrigerator until subsequent agarose gel electrophoresis (AGE). Primers sequences were shown in **Supplementary Table S2**.

### Bioinformatic analysis

For the purpose of exploring the correlation of MMP1 in breast cancer, we analyzed the expression level of MMP1 according to the TCGA datasets. In details, we verified the MMP1 expression level in subtypes of breast cancer by the UALCAN database (http://ualcan.path.uab.edu). To assess the potential clinical significance of MMP1 in breast cancer, we took advantage of the Kaplan-Meier Plotter database (http://kmplot.com/analysis/index.php). The potential binding sites of RUNX2 in the promoter region of MMP1 were predicted *via* utilizing the JASPAR database (http://jaspar.genereg.net/).

### Statistical analysis

Student’s t-test was used to analyze the differences between the two groups. All statistical analyses were performed with the GraphPad Prism (GraphPad Software) and SPSS 20.0 software as we described before (14). Only when P<0.05 was considered statistically significant differences. All data were shown as mean ± SD (n=3 independent experiments). *,P<0.05; **, P<0.01; ***, P<0.001; ****, P<0.0001.

## Results

### MDA-MB-231-Re cells manifested increased resistance and facilitated EMT compared with parental cells

**Figure 1:**
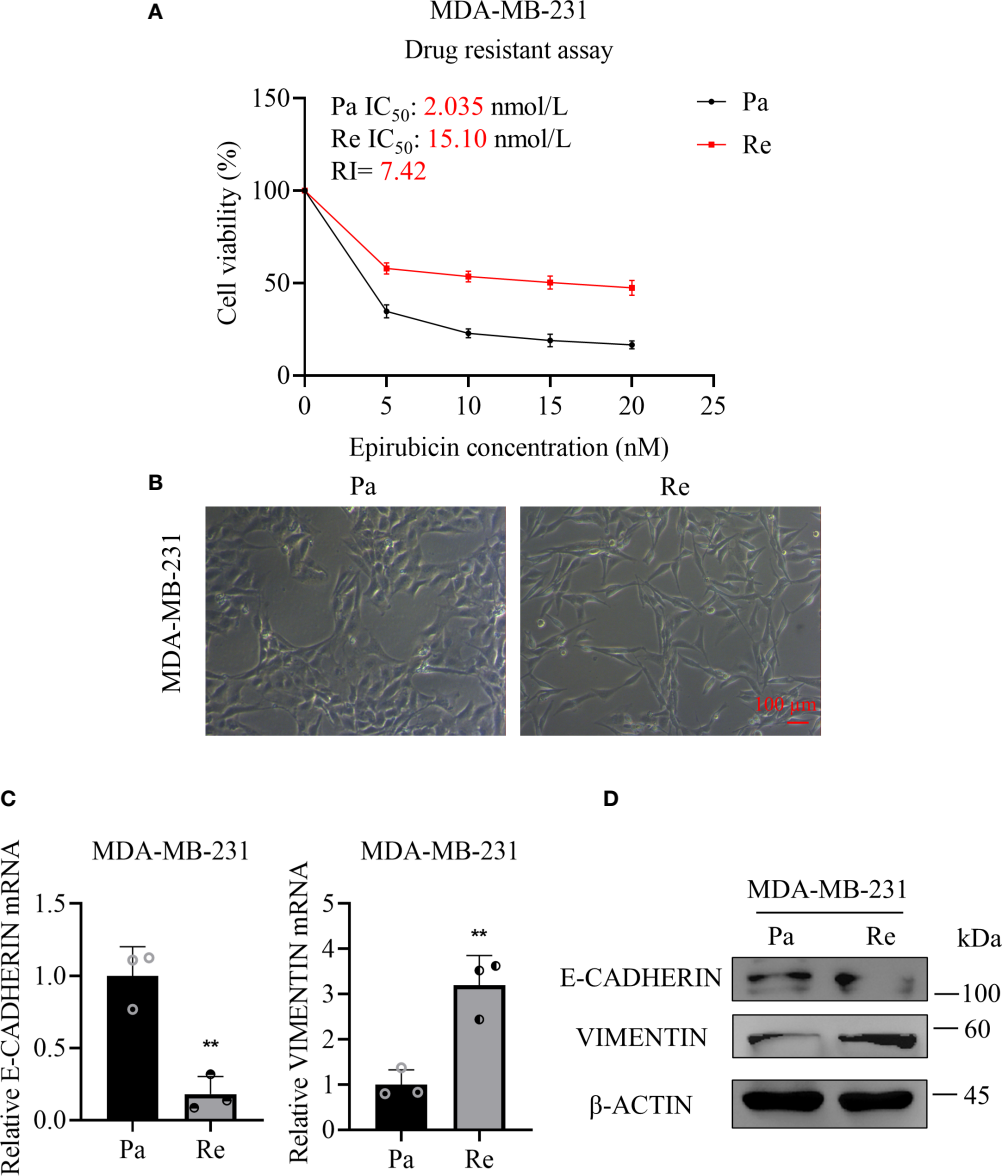
Drug resistance and cytomorphology of the drug-resistant triple negative breast cancer cell lines. **(A)** Cell viability detection after 24 h of Epirubicin treatment at various concentrations by CCK-8 assay. **(B)** Cell morphology was observed under a microscope. Scale bar =100 µm. **(C)** qRT-PCR assay for analyzing the expression of E-CADHERIN and VIMENTIN in MDA-MB-231-Re cells or MDA-MB-231-Pa cells. **(D)** Protein levels of E-CADHERIN, VIMENTIN and β-ACTIN were determined by western-blotting assay. All data were shown as mean ± SD (N=3 independent experiments). **P <0.01.

To generate the triple negative breast cancer resistant cell line MDA-MB-231-Re cells, we took advantage of the chronic-induced MDA-MB-231 cells for 8 months by step-up therapy (11). According to the CCK-8 values of the half of inhibition rate (IC_50_), we found the drug resistance of MDA-MB-231-Re cells was higher than that of MDA-MB-231 parental cells (15.10 nmol/l *vs.* 2.035 nmol/l). In details, the Epirubicin drug resistance index of MDA-MB-231-Re cells was 7.42-fold over that of the MDA-MB-231 cells (**Figure 1A**). Meanwhile, we noticed the visible differences in cytomorphology between the MDA-MB-231-Re cells and MDA-MB-231 parental cells (**Figure 1B**). Distinguish from the epithelial-like shape in the MDA-MB-231 group, MDA-MB-231-Re cells showed typical mesenchymal-like morphology instead (**Figure 1B**). To investigate whether MDA-MB-231-Re cells could facilitate EMT, qRT-PCR and western-blotting analyses were performed to detect the expression pattern of EMT-associated biomarkers in the MDA-MB-231-Re cells and MDA-MB-231 parental cells. As show in **Figures 1C, D**, the expression of epithelial-related biomarker E-CADHERIN was reduced, while the mesenchymal-related biomarker VIMENTIN was up-regulated. Taken together, the triple negative breast cancer resistant MDA-MB-231-Re cells revealed enhanced drug-resistance against Epirubicin and facilitated EMT compared to the parental MDA-MB-231 cells.

### RUNX2 was essential for migration and invasion of triple negative breast cancer cells

**Figure 2:**
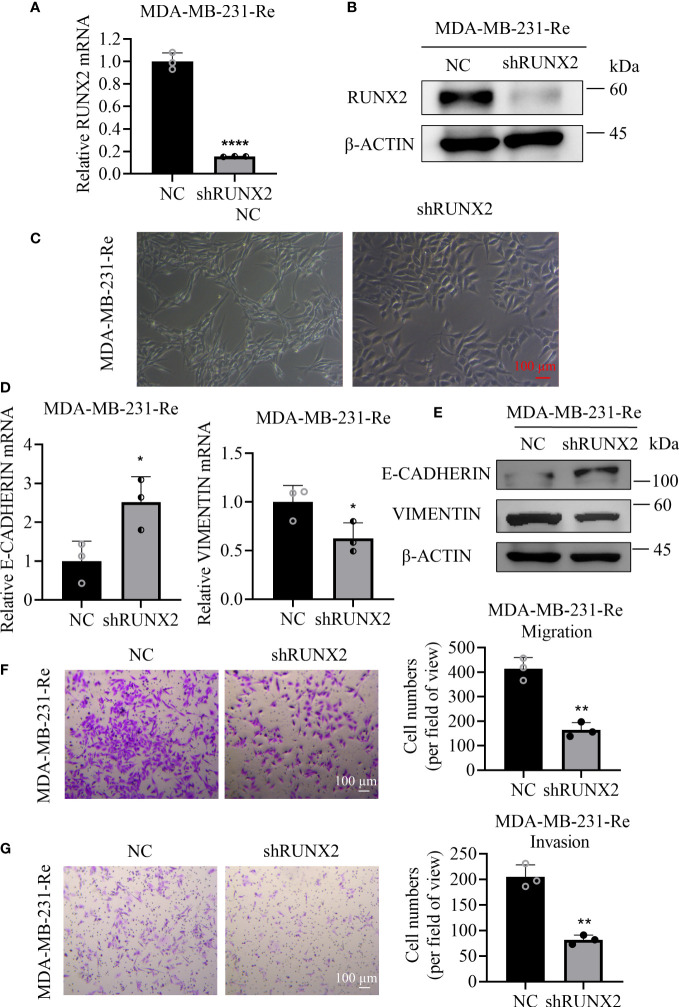
Influence of shRUNX2 on the migration and invasion of drug-resistant cells in triple negative breast cancer. **(A)** qRT-PCR analysis of RUNX2 expression in MDA-MB-231-Re cells with stable RUNX2 knockdown (shRUNX2) or Scramble-transduced negative control (NC). **(B)** Protein levels of RUNX2 and β-ACTIN were determined by western-blotting assay. **(C)** Cytomorphology of the shRUNX2 and NC MDA-MB-231-Re cells was observed under the microscope. Scale bar =100 µm. **(D)** qRT-PCR assay for analyzing the expression of E-CADHERIN and VIMENTIN in the shRUNX2 and NC MDA-MB-231-Re cells. **(E)** protein levels of E-CADHERIN, VIMENTIN and β-ACTIN were determined by western-blotting assay. **(F)** The migration ability of the shRUNX2 and NC MDA-MB-231-Re cells assessed by Transwell assay. Scale bar =100 um. **(G)** The invasion ability of the shRUNX2 and NC MDA-MB-231-Re cells assessed by Transwell assay. Scale bar =100 µm. All data were shown as mean ± SD (N=3 independent experiments). *P <0.05; **P <0.01; ****P<0.0001.

To assess the influence of RUNX2 in the migration and invasion of triple negative breast cancer, we transduced lentivirus-mediated shRUNX2 into MDA-MB-231-Re cells (denoted as shRUNX2) to knockdown the endogenous expression of RUNX2. By conducting qRT-PCR and western-blotting analyses, we found the expression of RUNX2 both at the transcription and translation level was consistently impaired in the shRUNX2 group compared with the negative control group (denoted as NC) (**Figures 2A, B**). Strikingly, MDA-MB-231-Re cells with stable RUNX2 knockdown restored the epithelial-like cytomorphology, which was distinguish from that in the NC group (**Figure 2C**).

Considering the switching action in orchestrating the cytomorphology, we supposed that RUNX2 might play a pivotal role in the progression of epithelial-mesenchymal transition (EMT). Therefore, qRT-PCR and western-blotting analyses were performed to verify the expression pattern of EMT-associated biomarkers in the shRUNX2 group and NC group, respectively. Intuitively, we found that RUNX2 knockdown increased the expression of E-CADHERIN, while reduced the expression of VIMENTIN (**Figures 2D, E**). Consistently, we took advantage of the transwell assay to evaluate the influence of RUNX2 knockdown upon MDA-MB-231-Re cells. Notably, the migration and invasion ability of the MDA-MB-231-Re cells with RUNX2 knockdown was consistently impaired (**Figures 2F, G**). Collectively, RUNX2 knockdown was adequate to inhibit EMT and reduce the *in vitro* migration and invasion in the triple negative breast cancer resistant MDA-MB-231-Re cells.

### Knockdown of RUNX2 inhibited the proliferation, drug resistance, and anchorage-independent growth in triple negative breast cancer cells

**Figure 3:**
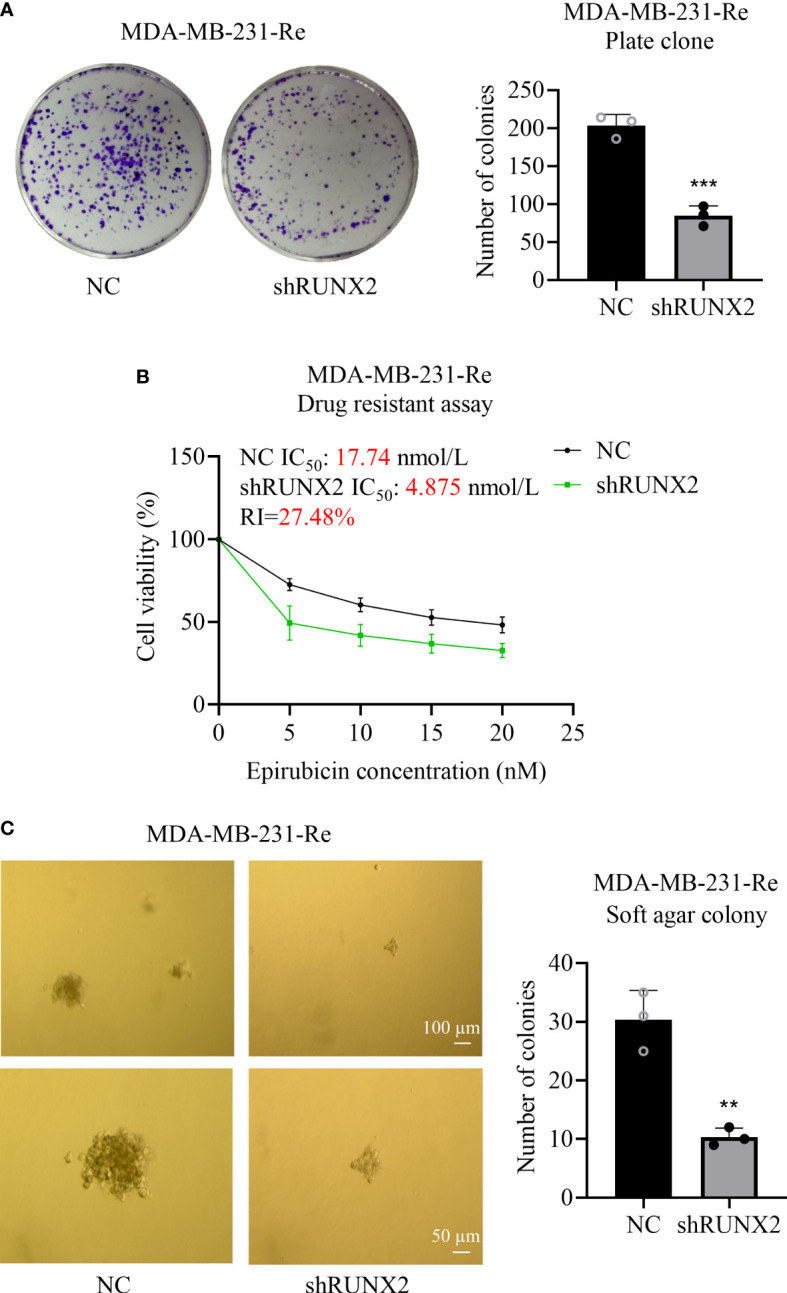
Influence of RUNX2 on the proliferation and resistance of drug-resistant cells in breast cancer. **(A)** Proliferative capacity of the shRUNX2 and NC MDA-MB-231-Re cells was determined by plate cloning assay. **(B)** Resistance to the shRUNX2 and NC MDA-MB-231-Re cells was determined by the CCK-8 assay. **(C)** The anchorage-independent growth capacity of the shRUNX2 and NC MDA-MB-231-Re cells was determined by a soft agar colony formation assay. Scale bar =100 µm, 50 µm. All data were shown as mean ± SD (N=3 independent experiments). **P<0.01; ***P<0.001.

Having clarified the inhibitory effect of RUNX2 knockdown upon cytomorphology as well as migration and invasion, we were next curious about the potential impact upon proliferation, drug resistance and anchorage-independent growth in MDA-MB-231-Re cells. With the aid of plate cloning assay, we found that fewer clones were formed in the shRUNX2 group compared to that in the NC group (**Figure 3A**). CCK-8 assay showed that the IC_50_ of MDA-MB-231-Re cells with shRUNX2 transfection was much lower than that in the NC group (4.875 nmol/l *vs.* 17.74nmol/l) as well, which indicated the weakened drug resistance according to the epirubicin resistance index (RI=27.48%) (**Figure 3B**). In consistence, soft agar colony formation assay further revealed the reduced anchorage-independent growth capacity of shRUNX2 cells compared to NC cells (**Figure 3C**). Taken together, the proliferation, drug resistance, and anchorage-independent growth in triple negative breast cancer MDA-MB-231-Re cells were consistently impaired by RUNX2 knockdown.

### Knockdown of RUNX2 efficaciously inhibited tumor growth in nude mouse

**Figure 4:**
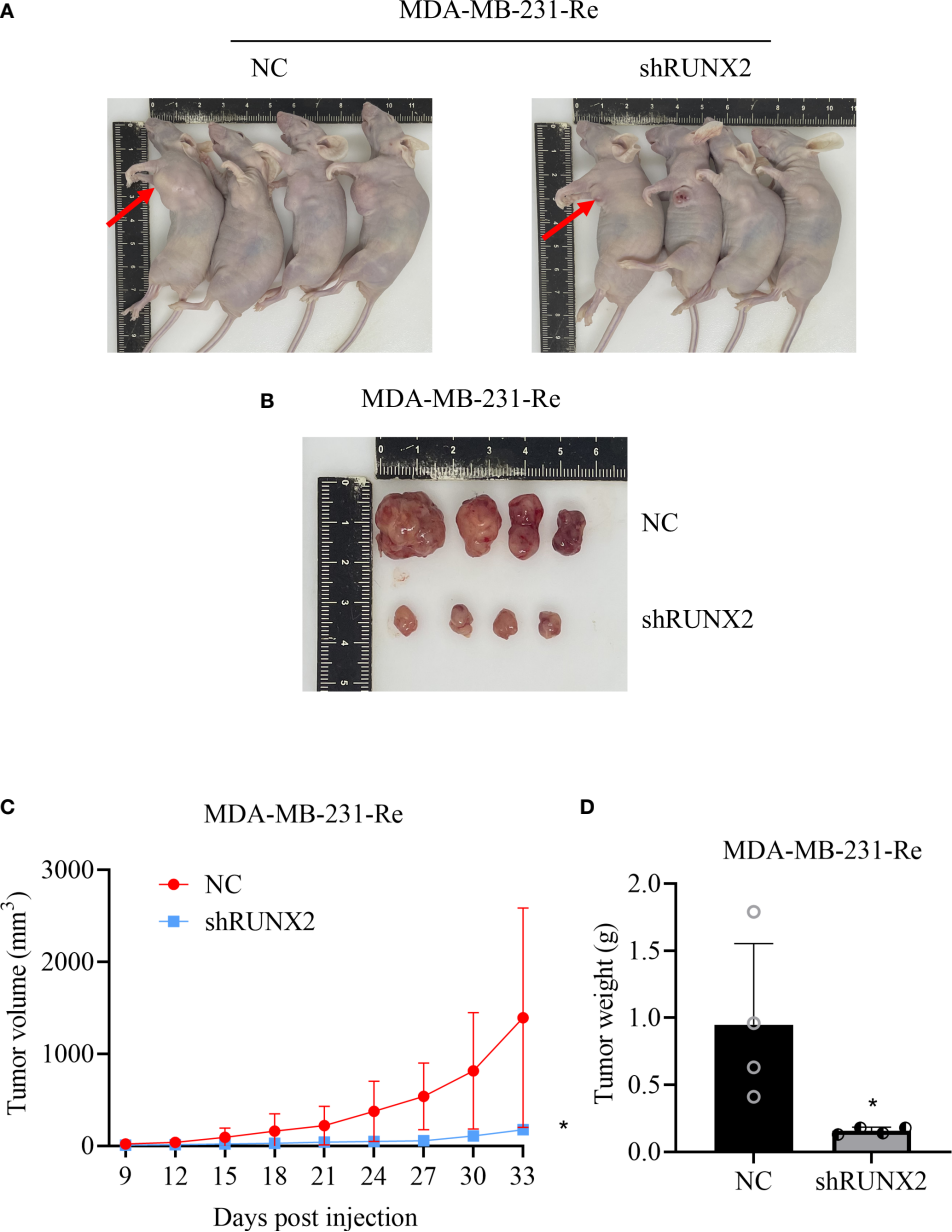
RUNX2 promoted the progression of breast cancer cells *in vivo*. **(A)** Image of *in vivo* subcutaneous neoplasia in BALB/c nude mice formed by the transplanted shRUNX2 and NC MDA-MB-231-Re cells. **(B)** Image of the tumor nodules formed by the transplanted shRUNX2 and NC MDA-MB-231-Re cells. **(C)** Tumor volume was measured in each group on the indicated days. **(D)** Tumors were removed after the last measurement on the 33rd day and weighed. All data were shown as mean ± SD (N=3 independent experiments). *P<0.05.

To further verify the oncogenic potential of RUNX2 *in vivo*, the aforementioned NC cells and shRUNX2 cells were subcutaneously injected into BALB/c nude mice for xenograft tumor formation. Distinguish from those formed in the NC group, tumor sizes in the shRUNX2 group were significantly decreased at day 33 after transplantation (**Figures 4A, B**). Furthermore, as shown by the spatio-temporal growth curves, the tumor volume and tumor growth rate of shRUNX2 cells were less than that of the NC cells (**Figure 4C**). Similarly, the tumor weight in the shRUNX2 group was also sharply declined when compared with the NC group (**Figure 4D**). Overall, our data indicated the crucial role of RUNX2 in tumorigenesis during triple negative breast cancer.

### shRUNX2 cells revealed discrepancy in gene expression profiling with NC cells

**Figure 5:**
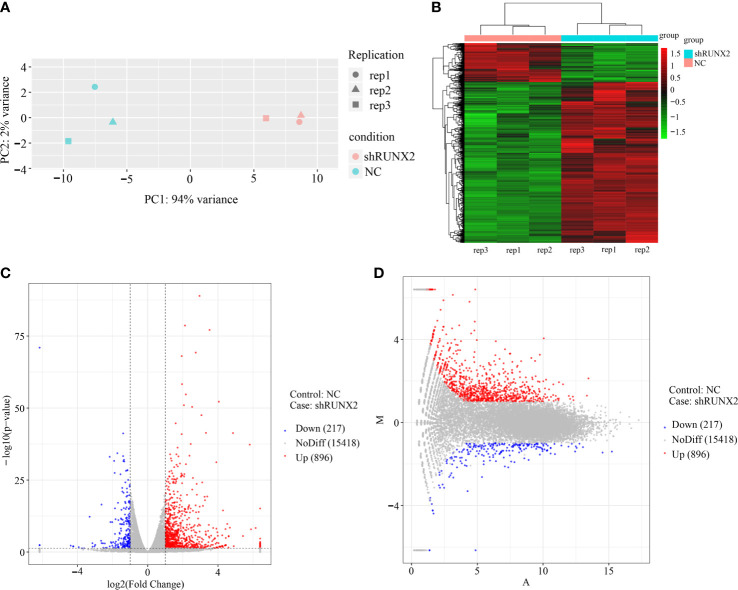
Gene expression profiling and DEGs in shRUNX2 and NC MDA-MB-231-Re cells. **(A)** Principal component analysis (PCA) of the three replications of shRUNX2 and NC MDA-MB- -231-Re cells. **(B)** Hierarchical cluster analysis of the DEGs in shRUNX2 and NC MDA-MB-231-Re cells. **(C, D)** Volcano plots **(C)** and MA plots **(D)** of the total genes expressed in shRUNX2 and NC MDA-MB-231-Re cells.

Having verified the cellular characteristics of shRUNX2 cells and NC cells for triple negative breast cancer, we next turned to dissect the underlying molecular mechanisms. Therewith, we took advantage of the RNA-SEQ analysis to explore the potent similarities and differences at the transcriptomic level. As shown by the principal component analysis (PCA), the three independent duplicate sample (rep1, 2, 3) in the shRUNX2 group (in pink) or the NC group (in green) were clustered, respectively (**Figure 5A**). Hierarchical cluster analysis further showed the discrepancy in the profiling of differentially expressed genes (DEGs) between the indicated two groups (**Figure 5B**). Furthermore, the volcano and MA plots intuitively revealed the distribution of the DEGs including 896 up-regulated genes and 217 down-regulated genes (|log2Fold Change|>1, P <0.05) (**Figures 5C, D**).

### MMP1 expression was significantly downregulated in shRUNX2 cells

**Figure 6:**
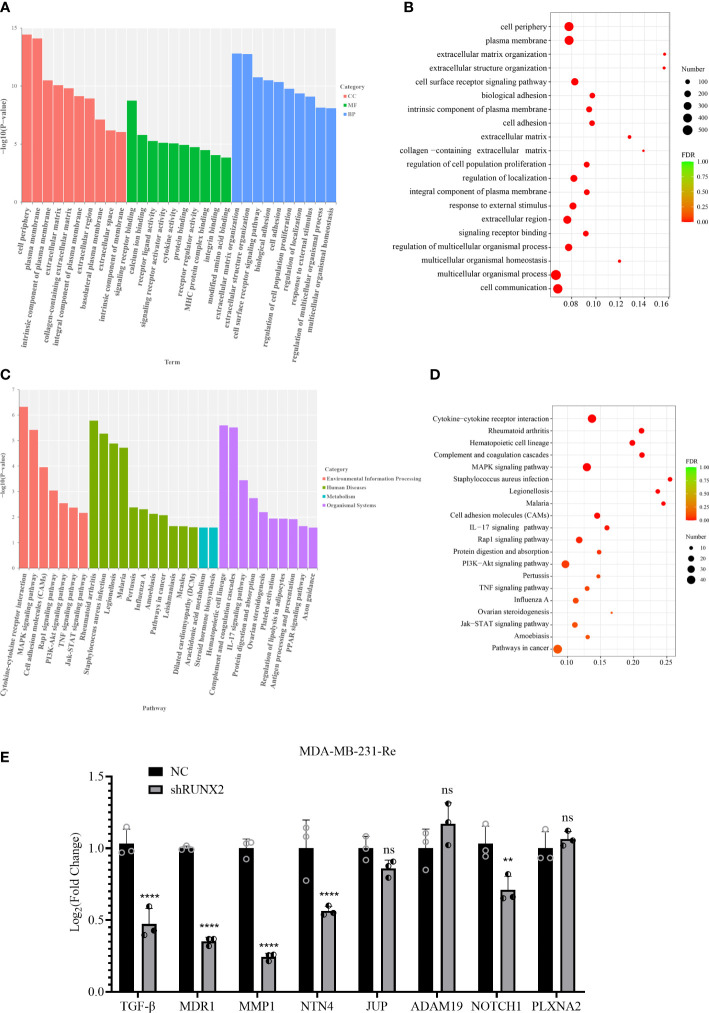
Function and signal prediction of the DEGs in TNBC. **(A, B)** Bar chart of the top 10 GO terms **(A)** and bubble plots of the top 20 GO terms **(B)** in each GO classification based on the enriched DEGs. **(C, D)** Bar chart **(C)** and bubble diagram **(D)** of the top 20 KEGG pathways based on the enriched DEGs. **(E)** qRT-PCR analysis of candidate gene expression in MDA-MB-231-Re cells in shRUNX2 and NC MDA-MB-231-Re cells. All data were shown as mean ± SD (N=3 independent samples). **P<0.01; ****P<0.0001; ns, not significant.

Aiming to clarify the biological significance of the DEGs, we performed top gene ontology (topGO) analysis and observed the enrichment of certain datasets such as extracellular matrix (cell components, short for CC), protein binding (molecular function, short for MF), and regulation of cell population proliferation (biological processes, short for BP) (**Figure 6A**). Meanwhile, EMT-associated subsets were enriched by conducting GO enrichment analysis, including extracellular region, cell periphery, cell communication, regulation of cell population proliferation (**Figure 6B**).

Subsequently, we took advantage of the Kyoto Encyclopedia of Genes and Genomes (KEGG) pathway database to further dissect the underlying variations accompanied with the DEGs. Notably, a certain number of signaling pathways associated with proliferation and tumorigenesis were enrich based on the -log10 (P value) such as PI3K-Akt, MAPK and Jak-STAT signaling pathways (**Figure 6C**). In consistence, KEGG pathway enrichment assay based on FDR values (FDR<0.001) further confirmed the specific enrichment of pathways in cancer, Jak-STAT, MAPK and PI3K-Akt signaling pathways, together with cell adhesion molecules(CAMs) (**Figure 6D**).

Therewith, we detected the mRNA expression pattern of extracellular matrix-, cell adhesion-, cell invasion and metastasis-associated DEGs in triple negative breast cancer MDA-MB-231-Re cells with stable NC or shRUNX2 transfection. Of the indicated candidates (e.g., TGF-β, MDR1, NTN4, NOTCH1), we noticed that the expression of MMP1 with the most downregulation in shRUNX2 cells when compared with the NC group (**Figure 6E**). Collectively, our data indicated the biological significance of DEGs were mainly related with EMT process (e.g., migration, invasion) and resistance. MMP1 might play a critical role in mediating the tumor-promoting effect of RUNX2 in triple negative breast cancer cells.

### MMP1 expression manifested positive correlation with poor prognosis of breast cancer

**Figure 7:**
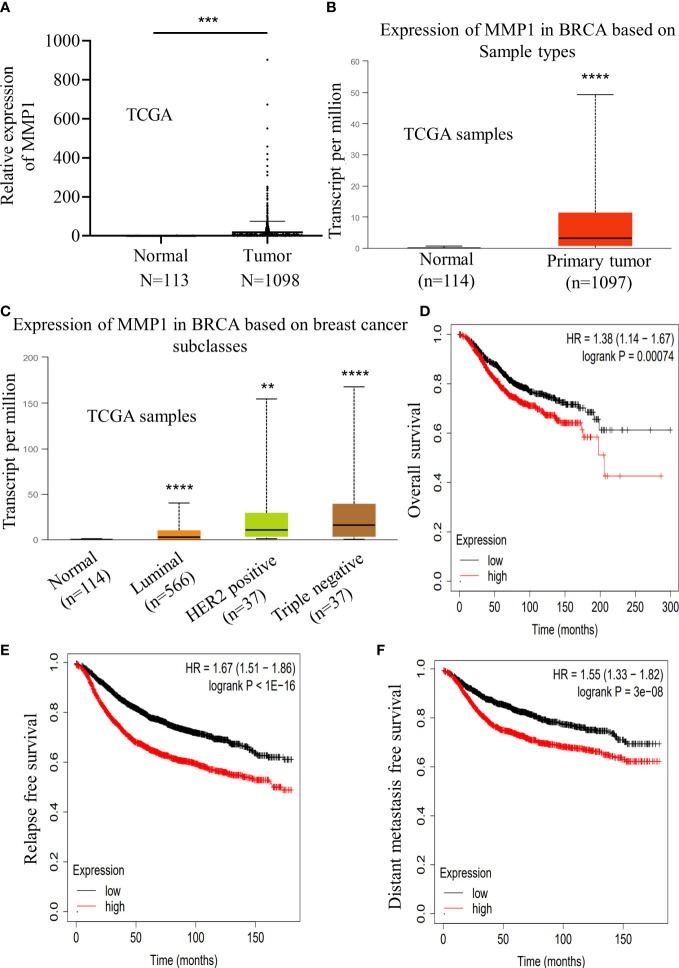
Increased expression of MMP1 indicated a poor prognosis in breast cancer. **(A)** Relative expression level of MMP1 in normal breast tissue samples (n=113) and breast cancer samples (n=1098) according to the TCGA database. **(B)** Relative expression level of MMP1 in normal breast tissue samples (n=114) and breast cancer samples (n=1097) according to the ualcan database. **(C)** Expression level of MMP1 in various types of breast cancer samples according to the ualcan database. **(D-F)** The relation of MMP1 expression levels according to the kmplot database and overall survival **(D)**, relapse free survival **(E)** and distant metastasis free survival **(F)** in breast cancer patients. **P<0.01; ***P<0.001; ****P<0.0001.

For the purpose of further exploring the correlation of MMP1 in breast cancer, we detected the MMP1 expression level in 1098 breast cancer samples and 113 normal breast tissues according to the TCGA datasets. Notably, we found breast cancer tissues with higher level of MMP1 expression over that in normal breast tissue, which was confirmed by the UALCAN database (http://ualcan.path.uab.edu) (**Figures 7A, B**). Furthermore, compared with other types of breast cancer, we noticed that triple negative breast cancer with the highest level of MMP1 expression (**Figure 7C**).

Meanwhile, we turn to assess the potential clinical significance in breast cancer. Based on the Kaplan-Meier Plotter database (http://kmplot.com/analysis/index.php), we found that MMP1 expression showed positive correlation with the overall survival, relapse free survival and distant metastasis free survival of patients with breast cancer (**Figures 7D–F**). Taken together, our data revealed the positive correlation of MMP1expression with poor prognosis of breast cancer.

### RUNX2 directly targeted MMP1 in triple negative breast cancer cells

**Figure 8:**
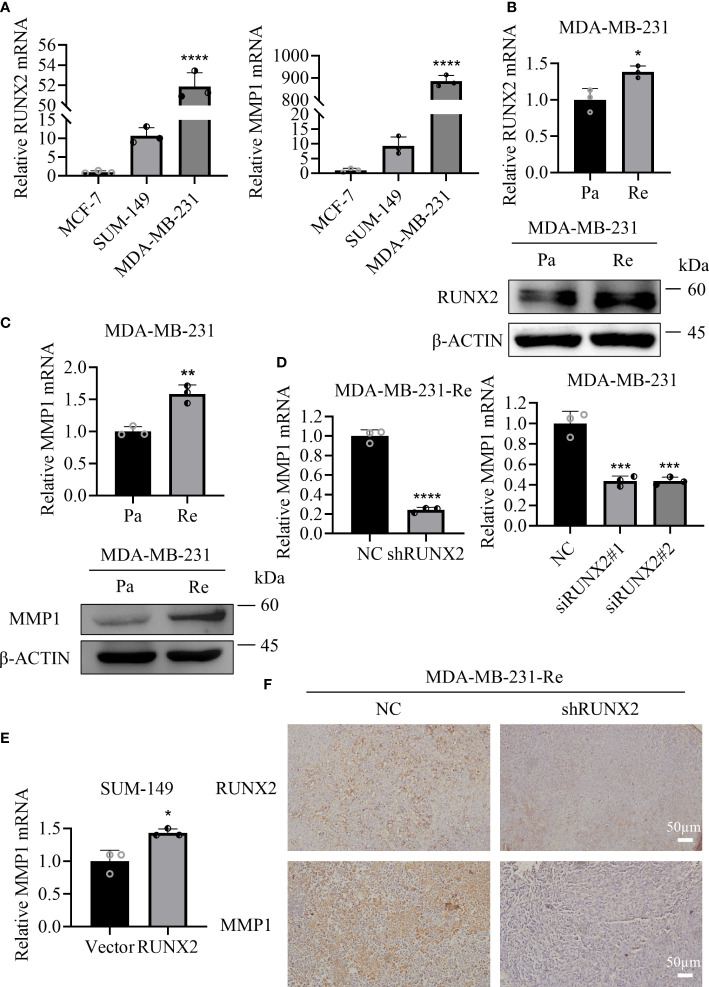
MMP1 and RUNX2 showed positively correlation in relative mRNA and protein expression. **(A)** qRT-PCR analysis of the expression of RUNX2 and MMP1 in various breast cancer cell lines. **(B, C)** qRT-PCR and western-blotting analyses of RUNX2 **(B)** and MMP1 **(C)** expression in MDA-MB-231-Re cells or MDA-MB-231-Pa cells. **(D, E)** qRT-PCR analysis of MMP1 expression in RUNX2 knockdown MDA-MB-231-Re and MDA-MB-231 cells **(D)** as well as the SUM-149 with RUNX2 overexpression **(E)**. **(F)** RUNX2 and MMP1 expression of tumor sections from each group were determined by immunohistochemistry. Scale bar =50 µm. *P <0.05; **P <0.01; ***P<0.001; ****P<0.0001.

To further illustrate the molecular mechanism of MMP1 and RUNX2 in TNBC, we initially examined the expression pattern of RUNX2 and MMP1 in the SUM-149 and MDA-MB-231 triple negative breast cancer cell line and the MCF-7 non-triple negative breast cancer cell line. Consistently, we found that the mRNA expression levels of RUNX2 and MMP1 revealed similar expression pattern, and in particular, those in triple negative breast cancer cell line were significantly higher than in non-triple negative breast cancer cell line (**Figure 8A**). Furthermore, by conducting qRT-PCR and western-blotting assays, we found the expression levels of MMP1 and RUNX2 both at transcription and translation level were higher in MDA-MB-231-Re cells compared with those in MDA-MB-231 cells (**Figures 8B, C**). Then, with the aid of shRNA- and siRNA- mediated knockdown of RUNX2, we observed the sharp decrease of MMP1 expression in MDA-MB-231-Re and MDA-MB-231 cells compared to the NC groups (**Figure 8D**). Simultaneously, the expression of MMP1 was significantly upregulated in SUM-149 breast cancer cells with RUNX2 overexpression (**Figure 8E**). Besides, the expression levels of RUNX2 and MMP1 in tumor sections from both groups of xenograft tumor were detected by immunohistochemical staining, which showed that they were obviously lower in tumors of the shRUNX2 group compared with those of the NC group (**Figure 8F**).

**Figure 9:**
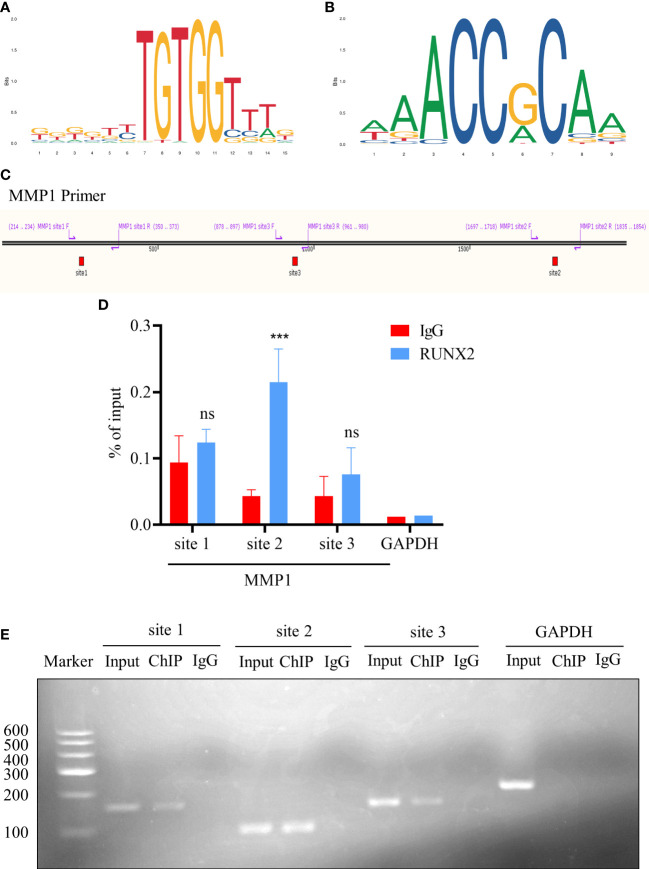
RUNX2 binds directly to the MMP1 promoter region. **(A, B)** The binding motif prediction of the RUNX2 according to the jaspar database. **(C)** Illustration of the three potential RUNX2 binding sites in the MMP1 promoter region. **(D)** ChIP-qRT-PCR analysis of the binding sites of RUNX2 in the MMP1 promoter region in MDA-MB-231-Re cells. **(E)** Agarose gel electrophoresis analysis of RUNX2 binding to the MMP1 promoter region in MDA-MB-231-Re cells. All data were shown as mean ± SD (N=3 independent samples). ***P<0.001; ns, not significant.

Having preliminarily verified the potential regulatory relationship between RUNX2 and MMP1, we assumed whether RUNX2 functions *via* direct activation of MMP1 expression. Therefore, we predicted the potential binding sites of RUNX2 in the promoter region of MMP1 according to the JASPAR database (http://jaspar.genereg.Net/) (**Figures 9A, B**; **Table 1**). Of the seven candidates, the top 3 binding sites (site 1, 2, 3) with the highest score were selected, for further identification (**Figure 9C**). Then, with the aid of chromatin immunoprecipitation (ChIP) and qRT-PCR analyses together with agarose gel electrophoresis, we found only site 2 was specifically enriched by RUNX2 in MDA-MB-231-Re cells with high level of RUNX2 expression (**Figures 9D, E**). This was finally verified by agarose gel electrophoresis (**Figure 9E**). Overall, these data collectively indicated the direct modulation of MMP1 by RUNX2 *via* specific binding motif within the promoter region.

**Table 1:** RUNX2 binding motif prediction in MMP1 promoter.

Site	Score	Start	End	Strand	Predicted sequence
site 1	9.80	247	261	+	GTGTTCTTTGGTCTC
site 2	7.08	1766	1780	–	GTAAACAGTGGTTTT
site 3	6.95	932	946	–	TGACTCTGGGGTCTT
site 4	6.80	1767	1775	+	AAACCACTG
site 5	4.86	374	382	–	TAACTACAA
site 6	4.50	1510	1518	+	GAACCTCAG
site 7	4.26	933	941	+	AGACCCCAG

## Discussion

**Figure 10:**
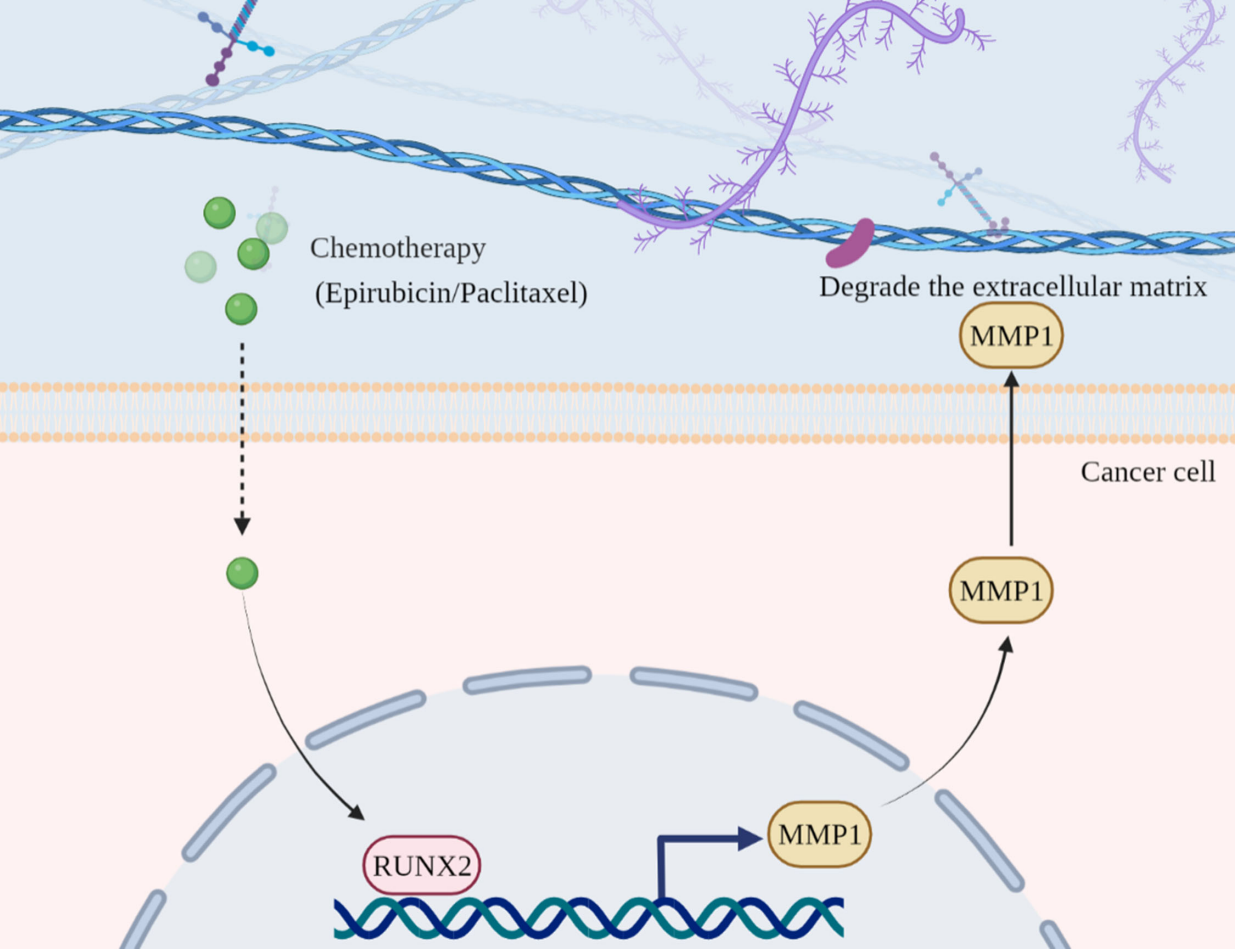
Illustration of the working model of RUNX2-MMP1 axis in TNBC. In chemotherapy drug-resistant TNBC, RUNX2 expression was upregulated against Epirubicin treatment. Then, RUNX2 was adequate to directly bind to the specific motif in the promoter region of MMP1 and thus activate MMP1 expression. Finally, the upregulated MMP1 functioned critically in the degradation of ECMs and the resultant modulation of proliferation, metastasis (e.g., migration, invasion) and drug resistance of TNBC.

The heterogeneous TNBC, characterized by the absence of progesterone receptor (PR) and HER2 expression, commonly results in poor prognosis due to the chemoresistance and infeasibility of targeted therapeutic approaches (3, 15). Therefore, despite chemotherapy serve as the “one size fits all” approach, yet the current treatment paradigm for TNBC administration is challenging and inopportune largely due to the deficiency of dissecting the clinical response and underlying molecular characteristics. Of note, state-of-the-art renewal has indicated the oncogenic role of RUNX2 in multiple cancer types (16–18), which the expression pattern and regulatory mechanism during chemoresistance in triple negative breast cancer are largely indistinct. For the purpose, we verified that RUNX2 knockdown was adequate to inhibit the *in vitro* proliferation, migration, invasion, and chemoresistance of resistant cells in triple negative breast cancer as well as the tumor growth in BALB/c nude mice. Furthermore, with the aid of transcriptomic analysis and ChIP-qRT-PCR assay, we identified that RUNX2/MMP1 axis functioned a crucial role in the malignant progression and poor prognosis in triple negative breast cancer (**Figure 10**).

RUNX2 has been recognized as the basic transcription factor for various physiological development (e.g., skeletal and osteogenic development) (6), and the concomitant pathological processes including cancer development and tumor bone metastasis (19). For instance, Guo et al. demonstrated the oncogenic role of RUNX2 during gastric carcinogenesis *via* YAP1 signaling (20). Wang and colleagues verified the promoting effect of Cbf-β in colorectal cancer progression in a RUNX2-dependent manner (21). Previously, we demonstrated that RUNX2 was adequate to activate TGF-β signaling pathway, and thus performed a pivotal role in TNBC Epirubicin-resistance by orchestrating the stemness, EMT and apoptosis of CD44^+^/CD24^-^ BCSCs (11). Notably, current literature has suggested the critical role of RUNX2 for malignant progression of breast cancer by modulating the CD44 ^+^/CD24^-^ breast cancer stem cells (22, 23). Instead, we not only verified the tumorigenic role of RUNX2 in TNBC progression and cancer chemoresistance both *in vitro* and *in vivo*, but also revealed the regulatory mechanism of RUNX2 *via* directly targeting the specific binding site in the promoter region of MMP1.

Matrix metalloproteinases (MMPs) are zinc-dependent endopeptidases, which are modulated by tissue inhibitors of metalloproteinases (TIMPs) and involved in the degradation of a certain number of proteins in the extracellular matrix (ECM) (24). To date, MMPs have been reported crucially in many processes, including embryonic development and tissue repair, tumor growth and metastasis (25). For instance, Liu et al. and Yang et al. ascertained the MMP1-dependent effect in hepatocellular carcinoma (HCC) progression *via* interacting with HuR and the metastasis of colorectal cancer *via* orchestrating the DDX3/YY1/MMP1/PI3K-AKT axis, respectively (26, 27). It’s noteworthy that MMP1 upregulation by promoter hypomethylation has been reported with enhanced tamoxifen resistance in breast cancer (28). In this study, by conducting multifaceted cellular and molecular analyses, we for the first time identified a specific binding motif in the promoter of MMP1 for the direct transmission of the tumorigenic effect of RUNX2, which would supply novel target candidates for drug development and clinical intervention decision against the proliferation, metastasis (e.g., migration, invasion) and drug resistance of TNBC (29, 30).

Overall, our data indicated the oncogenic effect and concomitant mechanism of the RUNX2-MMP1 axis in the modulation of the proliferation, migration, invasion and drug resistance in triple negative breast cancer drug-resistant cells *in vitro*, together with tumor growth of breast cancer cells *in vivo*, which would supply overwhelming new references for dissecting the pathogenesis of TNBC and facilitating the development of clinical remedies and novel therapeutic targets for triple negative breast cancer.

## Data availability statement

The datasets presented in this study can be found in online repositories. The names of the repository/repositories and accession number(s) can be found in the article/**Supplementary Material**.

## Ethics statement

The animal study was reviewed and approved by the Ethics Committee of the Experimental Animal Ethics Committee of Anhui Medical University.

## Author contributions

WS and FFL conceived and planned the study. WS, XDX, LW, and FXL performed the experiments. XDX, WW, XJX, and WL helped to prepare and analyze the samples. WS and XDX analyzed the data. WS and FFL wrote the manuscript. FFL, LZ, and DH completed critical revisions and proofread the manuscript. All authors contributed to the article and approved the submitted version.

## Funding

This work was supported by the National Natural Science Foundation of China (82173377, 81302319, 82260031), the Major Research Project of Education Department of Anhui Province (KJ2018ZD018), the Non-profit Central Research Institute Fund of Chinese Academy of Medical Sciences (2019PT320005), the 2021 Central-Guided Local Science and Technology Development Fund (ZYYDDFFZZZJ-1), the Anhui Provincial Natural Science Foundation of China (No.2008085MH299), the Key research and development Program of Anhui Province, China (No. 2022e07020012), the Fundamental Research Funds for the Central Universities (No. WK9110000086), Postdoctoral Research Funding of Anhui Province in 2019 (No. 2019B371).

## Conflict of interest

The authors declare that the research was conducted in the absence of any commercial or financial relationships that could be construed as a potential conflict of interest.

## Publisher’s note

All claims expressed in this article are solely those of the authors and do not necessarily represent those of their affiliated organizations, or those of the publisher, the editors and the reviewers. Any product that may be evaluated in this article, or claim that may be made by its manufacturer, is not guaranteed or endorsed by the publisher.
