# Supplementary material for: RUNX2 facilitates aggressiveness and chemoresistance of triple negative breast cancer cells via activating MMP1
Source: Front Oncol. 2022 Nov 18;12:996080. doi: 10.3389/fonc.2022.996080 (PMC9724742; doi:10.3389/fonc.2022.996080)
Supplement: Supplementary file 1 [file Table_1.docx]

***Supplementary Material***

**Supplementary table S1**

| primer | sequence |
| --- | --- |
| RUNX2 Forward | CCGGAATGCCTCTGCTGTTATGA |
| RUNX2 Reverse | ACTGAGGCGGTCAGAGAACAAACT |
| TGF-β Forward | CAATTCCTGGCGATACCTCAG |
| TGF-β Reverse | GCACAACTCCGGTGACATCAA |
| MDR1 Forward | GGGATGGTCAGTGTTGATGGA |
| MDR1 Reverse | GCTATCGTGGTGGCAAACAATA |
| MMP1 Forward | GAGCTCAACTTCCGGGTAGA |
| MMP1 Reverse | CCCAAAAGCGTGTGACAGTA |
| NTN4 Forward | GTACTTTGCGACTAACTGCTCC |
| NTN4 Reverse | TCCAGTGCATGGAAAAGGACT |
| JUP Forward | GTGGAGTCGGTCCTGTTCTATG |
| JUP Reverse | GTTACGCATGATCTGCACGAG |
| ADAM19 Forward | GGATGGACAAGAGGAAGTGAGG |
| ADAM19 Reverse | TGAGCTCAGCTTTGAGTGGA |
| PLXNA2 Forward | TCCATGACACAGATGTGCGG |
| PLXNA2 Reverse | TCAAACACGAACTGGGGGTT |
| NOTCH1 Forward | TGAATGGCGGGAAGTGTGAA |
| NOTCH1 Reverse | ACTTGTACTCCGTCAGCGTG |
| GAPDH Forward | CTCATTTCCTGGTATGACAACG |
| GAPDH Reverse | TTACTCCTTGGAGGCCATGT |

**Supplementary table S2**

| primer | sequence |
| --- | --- |
| MMP1 site1 Forward | TCTTTGTCTGTGCTGGAGTCA |
| MMP1 site1 Reverse | TTTCCTCATCTAAGTGGCATAACA |
| MMP1 site2 Forward | GCACCAAGGAGCGAAGATAG |
| MMP1 site2 Reverse | GAGAAGACCCCTCATCCACA |
| MMP1 site3 Forward | CCATGGAGTACTCTTTGACCTG |
| MMP1 site3 Reverse | TTGGAATCACTTGGTGTTGC |
| GAPDH Forward | AAAAGCGGGGAGAAAGTAGG |
| GAPDH Reverse | AAGAAGATGCGGCTGACTGT |
